# Schwannoma of the Sigmoid Colon: A Rare Case

**DOI:** 10.7759/cureus.53140

**Published:** 2024-01-29

**Authors:** Bushra A Zainaldeen, Amr S Alaus, Mariam AlKooheji, Jaffar Alkhuzaie, Safa Alshaikh

**Affiliations:** 1 Department of Surgery, Salmaniya Medical Complex, Manama, BHR; 2 Department of Pathology, Salmaniya Medical Complex, Manama, BHR

**Keywords:** peripheral nerve sheath tumor, gastrointestinal, colon, sigmoid, schwannoma

## Abstract

Schwannoma is a type of peripheral nerve sheath tumor that is often found in the head and neck. Schwannomas in the digestive system, particularly the colon and rectum, are exceptionally rare, and they are mostly non-malignant and asymptomatic although sometimes patients can present with symptoms similar to those observed in patients with other gastrointestinal tumors like abdominal pain, fullness, nausea, vomiting, and change in bowel habits. For diagnosis and treatment, surgical resection along with biopsy is the gold standard. In this paper, we describe a rare case of sigmoid schwannoma that was successfully treated in our department by surgical resection.

## Introduction

Schwannomas are usually slow-growing, non-malignant tumors that emerge from peripheral nerves derived from native Schwann cells [1]. The presence of these tumors within the gastrointestinal tract is rare, constituting roughly 2-6% of all non-epithelial tumors [2]. Their distribution follows a descending pattern, with the stomach being the most common site (83%), succeeded by the small bowel (12%), and lastly, the colon and rectum [2]. Gastrointestinal stromal tumors (GISTs), neuroendocrine tumors (NETs), leiomyoma, and leiomyosarcoma are included in the differential diagnoses for gastrointestinal schwannoma [2,3]. However, even with the use of multiple diagnostic modalities, it may be difficult to diagnose them preoperatively [3]. A conclusive pathological examination of the surgical specimen is essential for establishing the diagnosis [2]. Here, we present a case of a 64-year-old male who was diagnosed with schwannoma of the sigmoid colon.

## Case presentation

A 64-year-old male patient, with a known case of varicose veins, primary hypertension, and mixed hyperlipidemia for one and a half years, who has been on a daily regimen of perindopril arginine 5 mg + indapamide 1.25 mg coated tablet and atorvastatin 20 mg coated tablet at bedtime, presented to the accident and emergency department with a one-week history of severe, constant central upper abdominal pain that was associated with nausea and multiple episodes of vomiting. The patient gave a history of on-and-off constipation over the last few months and left lower quadrant pain for the past year and a half. Surgical history was significant for hemorrhoidectomy.

On examination, vital signs were within normal range, and the patient was fully oriented. Abdominal examination revealed a soft, lax abdomen with a localized area of tenderness in the left lower quadrant. Laboratory investigations did not reveal any significant findings, including hemoglobin concentration, which was 14.7 g/dL (normal range: 12.0-14.5 g/dL).

**Figure 1:**
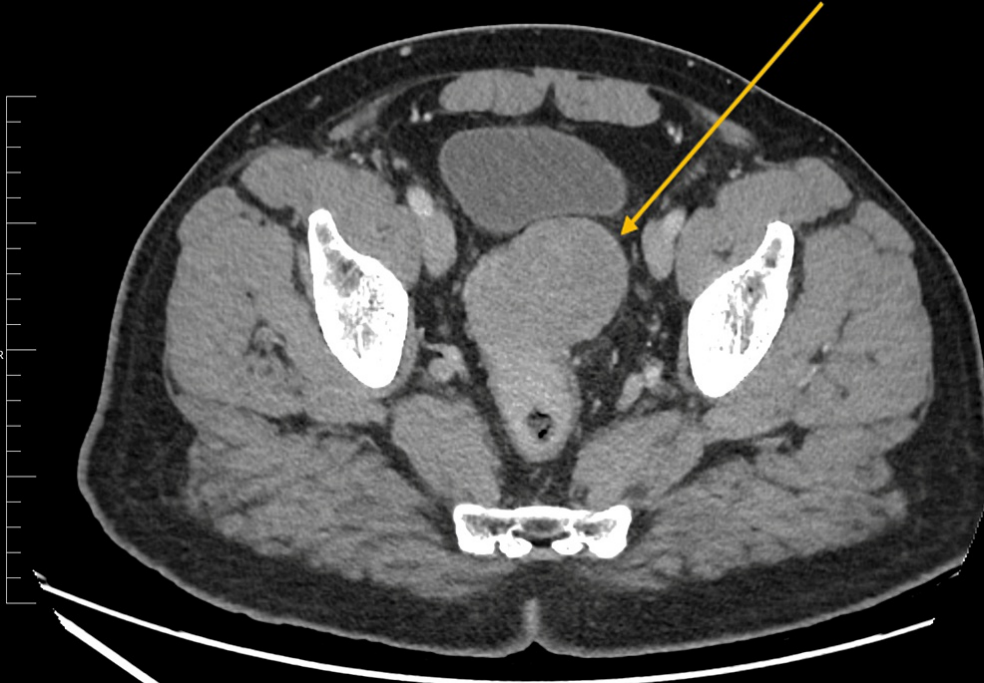
Axial abdominal CT scan showing a well-defined mass with heterogeneous enhancement arising from the third part of the rectum.

The CT scan of the abdomen revealed a well-defined heterogeneous mass measuring 5.5 x 5 x 5 cm in size. The mass was located in the third part (high part) of the rectum, 9 cm away from the anorectal angle. The mass appeared to be arising from the anterior rectal wall and was associated with multiple adjacent prominent lymph nodes (Figure 1).

The patient was discharged from the emergency department with analgesics and an outpatient appointment in the surgical clinic. The patient was scheduled for a gastro-colonoscopy at the clinic. The gastro-colonoscopy revealed a large rectal polypoid mass, located 10 cm from the anal verge. A biopsy was taken, which showed non-specific inflammation.

**Figure 2:**
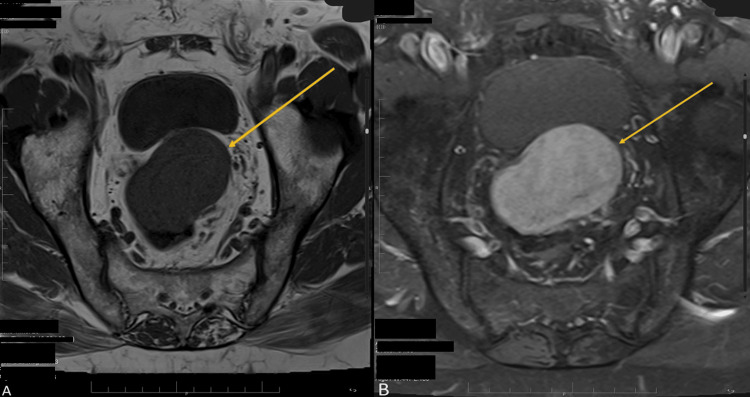
Abdominal MRI (T1-weighted images). (A) A T1 hypointense mural rectosigmoid mass. (B) A heterogeneous enhancement with areas of cystic changes on the post-contrast study.

**Figure 3:**
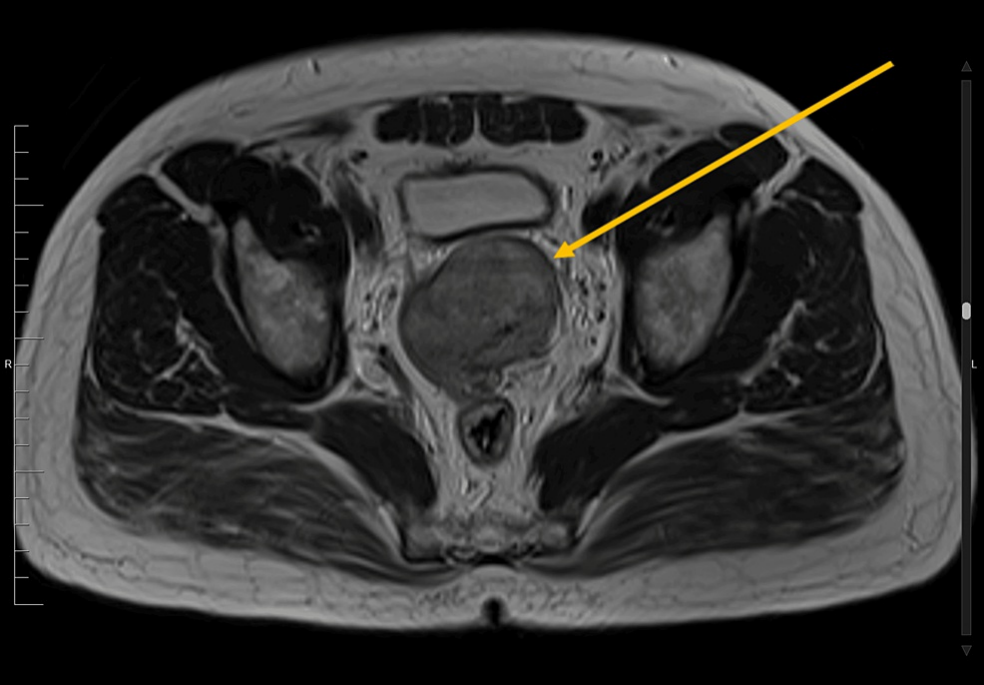
Abdominal MRI: T2-weighted image shows a T2 hyperintense mural rectosigmoid mass.

An MRI of the abdomen and pelvis was performed, which showed a large well-defined polypoidal exophytic soft tissue lesion. The lesion measured 5.3 x 5 x 5.2 cm and originated from the upper rectum, approximately 13 cm proximal to the anal verge. No associated lymphadenopathy was observed. The findings suggest a GIST as the most likely diagnosis (Figures 2, 3).

The patient underwent an anorectal examination under general anesthesia. A rectal biopsy was taken through rigid sigmoidoscopy, which revealed mild chronic inflammation.

A repeated colonoscopy was performed, which revealed a rectal bulge at 15-18 cm from the anal verge, causing extra luminal compression. A biopsy was taken, which showed colonic mucosa with a few dilated crypts and non-specific inflammation. No dysplastic changes or infiltration were noted. A repeated CT scan and MRI demonstrated the same findings as the previous imaging.

**Figure 4:**
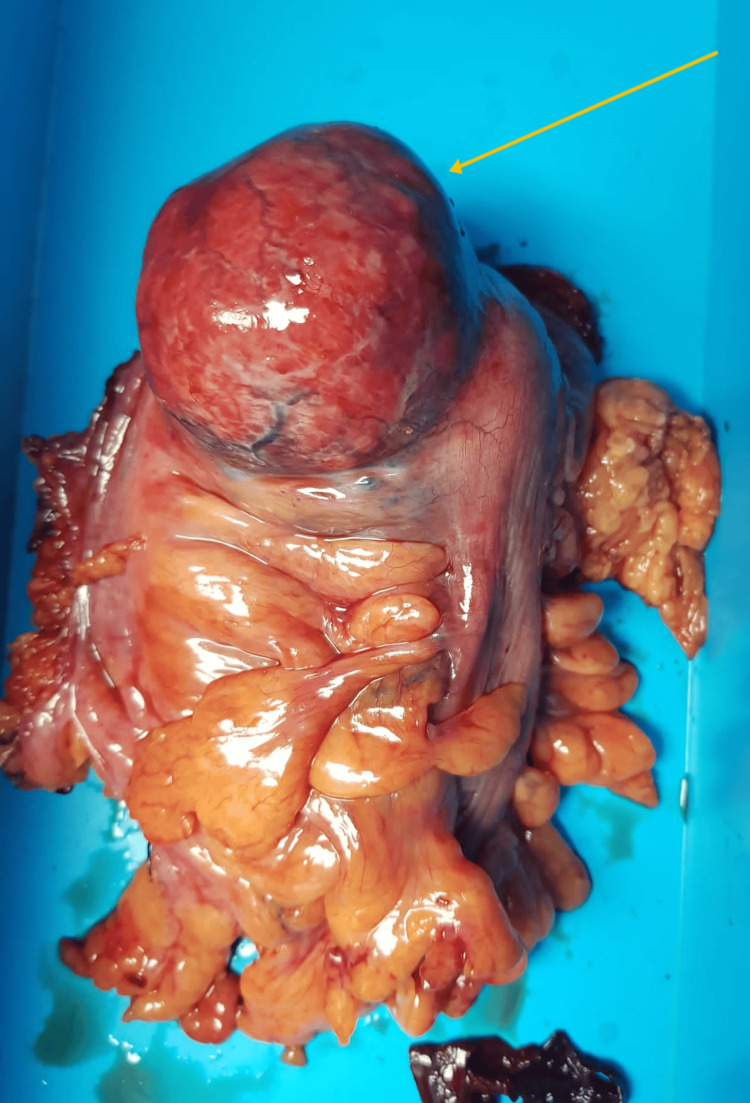
Gross picture showing the exophytic mass in the distal sigmoid.

The decision was made to perform an exploratory laparotomy. Intra-operative findings revealed a 10 x 5 cm exophytic bilobed mass in the distal sigmoid (Figure 4), with no mesenteric lymph nodes observed. As a result, the patient underwent a sigmoidectomy and stoma creation.

**Figure 5:**
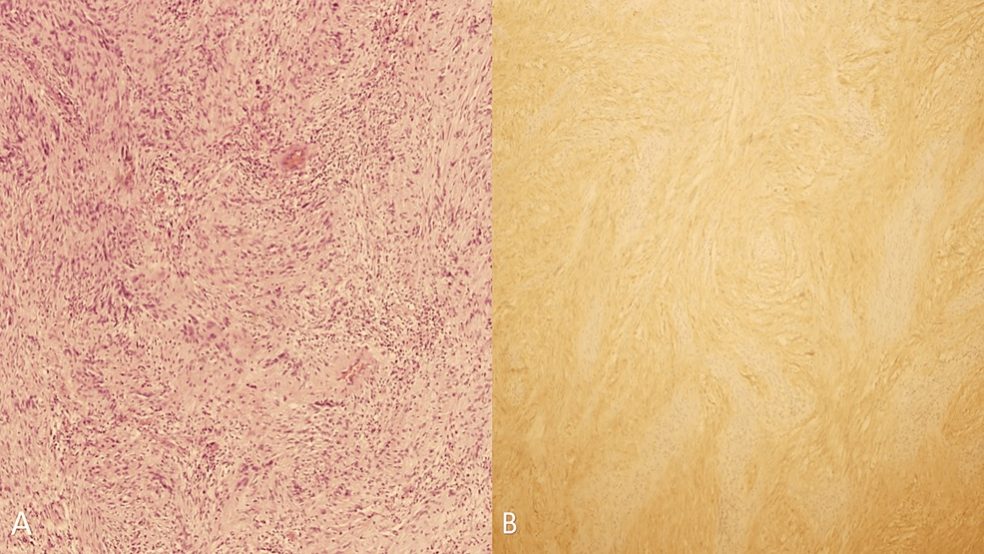
(A) Nuclear palisading is common in the cellular areas. The “nuclear-free zones” that lie between the nuclear palisades are called Verocay bodies (hematoxylin & eosin stain, 20×). (B) The tumor cells are immunoreactive for S100.

The final histopathology report indicated that the mass was a schwannoma (Figure 5).

Six months after Hartmann's procedure, the patient underwent a closure of colostomy with restoration of bowel continuity. The procedure was uneventful, and the patient was discharged on postoperative day 11 in good health.

## Discussion

Peripheral nerve sheath tumors represent infrequent occurrences among soft tissue tumors, with schwannomas emerging as the predominant lesions. These benign, encapsulated tumors originate from the Schwann cells of the peripheral nerve sheath, actively producing myelin [1]. The term "neurinomas" was initially coined by Uruguayan neuro-pathologist Jose Verocay in 1908, marking the early identification of these tumors [1,4]. Subsequently, in 1968, Harkin and Reed introduced the term "schwannoma" to characterize these well-defined, perineural lesions [1].

A colorectal schwannoma is an exceptionally uncommon tumor and represents the least common site for a gastrointestinal schwannoma [2,5,6]. Schwannomas are commonly observed in the stomach and small intestine, with infrequent occurrences in the colon or rectum [7]. Schwannomas in the colorectal region exhibit a higher prevalence in the right colon and cecum, with the sigmoid colon, rectum, left colon, and least commonly, the transverse colon following in descending order [2].

In 1988, Daimaru et al. were the first to define nerve sheath tumors in the gastrointestinal tract, suggesting the term "benign schwannoma of the GI tract" for these tumors [8,9]. Typically diagnosed in individuals in their sixth decade of life, these tumors show similar incidence rates for both males and females [2,8-11]. While these tumors are typically identified as solitary lesions, there is a possibility of them occurring at various locations throughout the body [1].

Because they are frequently asymptomatic, the majority of them are discovered by chance during screening endoscopy or imaging investigations. Nonetheless, like other gastrointestinal tumors, they may manifest symptoms like abdominal pain, tenesmus, rectal bleeding, or melena [8,12].

While differential diagnoses for gastrointestinal schwannomas encompass GISTs, NETs, leiomyoma, and leiomyosarcoma, their preoperative diagnosis can be challenging despite employing various diagnostic modalities [3]. Bohlok et al.'s study revealed that 24% of colorectal schwannomas received a preoperative diagnosis [2,8]. Confirming the diagnosis of schwannoma is crucial, considering that alternative differentials such as GISTs possess a significantly higher malignant potential and potential risk for recurrence [12].

Exophytic masses with homogeneous enhancement are typically observed on CT scans, while cystic change, necrosis, or calcification within tumors are rare [8,13]. Endoscopically, they often manifest as submucosal tumors with smooth mucosa or alternatively with mucosal ulceration [2,8,12].

Similar to other mesenchymal tumors, mucosal biopsy tends to be inconclusive, and the definitive diagnosis relies on the immunohistopathologic examination of the operative specimen [2]. Distinctive features like nuclear atypia, positive immunostaining for S100 protein, and lymphoid cuffing set gastrointestinal tract schwannoma apart from other spindle-cell stromal tumors originating from smooth muscle [8,9].

There are two described histological growth patterns: Antoni A, characterized by densely arranged fusiform cells forming Verocay bodies in palisades. In Antoni B, the fusiform cells are scattered in a loose arrangement, featuring nuclei that are either rounded or elongated, accompanied by abundant myxoid stroma and xanthomatous histiocytes. Identifying these patterns has proven valuable in histologically identifying schwannomas [2,6].

Opting for complete surgical resection with free resection margins stands as the optimal therapeutic choice [2,8,12]. The recurrence of tumors primarily stems from incomplete surgical resection, where margins are inadequately addressed [8,13].

The overall prognosis is usually optimistic since the majority of GI schwannomas exhibit a benign nature with low malignant potential [2,8,12]. Colorectal schwannomas, specifically, are documented as benign in over 98% of cases, showcasing a low mitotic rate, the absence of atypical mitotic figures, and nuclear hyperpigmentation [2]. According to Bohlok et al.'s study, only three (3.1%) out of 93 cases of colorectal schwannomas were identified as malignant [2,8].

## Conclusions

The sigmoid colon is an uncommon location for the development of schwannoma. However, it is mostly a benign neoplasm. Sigmoid schwannoma is difficult to diagnose due to its non-specific clinical picture, as well as colonoscopic and CT findings. Biopsies from the mass are inconclusive. The final diagnosis is made by pathological examination of the operative specimen, which has a positive immunostaining for S100 protein. The advised treatment involves surgically removing the affected area with a wide margin.
